# Synthesis and Anti–*Mycobacterium tuberculosis* Evaluation of Aza-Stilbene Derivatives

**DOI:** 10.1100/tsw.2011.110

**Published:** 2011-05-26

**Authors:** Fernando R. Pavan, Gustavo Senra G. de Carvalho, Adilson D. da Silva, Clarice Q. F. Leite

**Affiliations:** ^1^ Faculdade de Ciências Farmacêuticas, Universidade Estadual Paulista, Araraquara, SP, Brazil; ^2^ Departamento de Química, ICE, Universidade Federal de Juiz de Fora, Juiz de Fora, MG, Brazil

**Keywords:** tuberculosis, new drugs, stilbenes, aza-stilbene derivatives

## Abstract

Tuberculosis (TB) is a truly global disease, found in every country on earth. One-third of humanity, over 2 billion people, carry the bacillus that causes TB and 2 million people die of the disease each year. Despite that, no new specific drug against *Mycobacterium tuberculosis* has been developed since the 1960s. There are several candidates for new anti-TB agents, but none proven clinically effective. Stilbenes are compounds found in numerous medicinal plants and food products with some known biological and even antimycobacterial activity. This paper describes the synthesis and the anti–*M. tuberculosis* activity of eight stilbene analogues. The synthesis and characterization of these compounds are shown, and the results compared with one “first”-line drug used in current therapy.

### INTRODUCTION

Tuberculosis (TB) has re-emerged as one of the leading causes of death in the world, reaching a million deaths annually. Mortality rates decreased globally in 2007, with 1.3 million HIV-negative TB patients dying in 2007 and 456,000 deaths among individuals infected with both TB and HIV[1]. However, multidrug resistant (MDR) TB, extensive drug resistant (XDR) TB, and TB/HIV are stifling attempts to control TB and causing suffering, death, and impoverishment worldwide[2]. Rifampin, discovered over 40 years ago, represents the last novel class of antibiotics introduced for the first-line treatment of TB. Drugs in this class are part of a 6-month regimen that is ineffective against MDR and XDR TB, and difficult to use with many antiretroviral drugs[3]. Thus, there is an urgent need to develop new therapies for TB, to reduce the duration of treatment, and to provide a more effective therapy for resistant and latent TB infection[4].

Stilbenes, such as resveratrol, piceatannol, and pinosylvin, are compounds found in numerous medicinal plants and food products (Fig. 1)[5,6,7]. The natural stilbene most relevant and more described in the literature is resveratrol, which was first isolated from Chinese and Japanese medicinal plants in 1963[8]. In 1992, this compound was postulated to explain some of the cardioprotective effects of red wine (the so-called "French paradox")[9,10,11].

**Resveratrol**: $R_4'$ = OH, $R_3'$ = H, $R_3 = R_5$ = OH
**Piceatannol**: $R_3' = R_4' = R_3 = R_5$ = OH
**Pinosylvin**: $R_3 = R_5$ = OH

**FIGURE 1.** Structure of the most common natural stilbenes.

Since then, dozens of studies have indicated that resveratrol plays an important role in preventing or slowing the progression of many diseases and illnesses, such as inflammation[12,13,14,15], cancer[6,7,14], and heart diseases[8,16]. Recently, additional properties of resveratrol have been documented, such as radical scavenging, antioxidant activity[15,17], neuroprotection[15,18], antiviral activity[15,19], antibacterial activity[20,21,22], antitubercular activity[23,24,25], and others[6,8,26,27].

On the other hand, little attention has been given to bioisostere analogues of stilbenes. Based on the concept of bioisosterism[28], the basic skeleton of trans-stilbene was modified by replacement of the central C=C linkage with a C=N double bond. This modification was carried out by the reaction of an aldehyde and an amino acid through an imine formation reaction (Fig. 2).

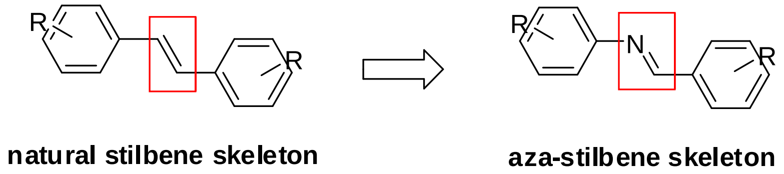

**FIGURE 2.** Classical bioisosterism approach.

It has recently been reported that a natural stilbene showed good results against *M. bovis*[23].

Due to our expertise, we have worked on and reported on new natural and synthetic (organic and inorganic) compounds with antimycobacterial activities[29,30,31,32,33,34,35,36]. In this paper, we propose to study the anti–*M. tuberculosis* (MTB) activity of this class of molecules (aza-stilbene derivatives) and to describe the synthesis of these stilbene analogues.

### MATERIAL AND METHODS

#### Chemistry

The typical procedure for the preparation of aza-stilbene derivatives was carried out. All compounds were synthesized by a facile and efficient imine formation reaction. For this, a mixture of corresponding aromatic aldehyde (5.50 mmol) and 4-hydroxy-aniline (5.00 mmol) was stirred in anhydrous ethanol (10 mL) at room temperature for 12 h. The crystalline powder formed in precipitation was collected by filtration, washed with cold EtOH, and dried in a vacuum oven to give the desired product. When necessary, the compounds were crystallized in acetonitrile. The NMR of $^{1}$H and $^{13}$C spectra were collected in a Bruker Avance DRX300; IR spectra were registered in a Bomen-FTIR MB-102 spectrometer. The most important spectroscopic information is shown in Table 1.

**TABLE 1**
**Spectral Data of Aza-Stilbene Derivatives**

| Compound | δ C*H*=N | δ *C*=N | $\bar{\nu}$ C=N | M.P. (ºC) | Yield (%) |
|---|---|---|---|---|---|
| 1 | 8.61 (s, 1H) | 157.1 | 1624 | 189.3 | 62 |
| 2 | 8.43 (s, 1H) | 157.1 | 1607 | 203.7 | 53 |
| 3 | 8.89 (s, 1H) | 157.1 | 1616 | 141.4 | 55 |
| 4 | 8.42 (s, 1H) | 156.9 | 1615 | 197.0 | 60 |
| 5 | 8.51 (s, 1H) | 156.6 | 1609 | 189.0 | 61 |
| 6 | 8.51 (s, 1H) | 157.0 | 1624 | 185.4 | 68 |
| 7 | 8.38 (s, 1H) | 156.9 | 1610 | 182,7 | 75 |
| 8 | 8.73 (s, 1H) | 157.3 | 1624 | 172.1 | 76 |

The NMR experiments were performed at 300 MHz for $^{1}$H and 75 MHz for $^{13}$C in DMSO-$d_6$ (ppm), and IR experiments were performed at KBr support (cm$^{-1}$).

### Biology

An anti-MTB activity assay was performed. The anti-MTB activity of the compounds was determined by the resazurin microtiter assay (REMA)[37]. Stock solutions of the test compounds were prepared in dimethyl sulfoxide (DMSO) and diluted in Middlebrook 7H9 broth (Difco Laboratories, Detroit, MI, USA), supplemented with oleic acid, albumin, dextrose, and catalase (OADC enrichment; BBL/Becton Dickinson, Sparks, MD, USA), to obtain final drug concentration ranges from 0.15 to 250 µg/mL. The serial dilutions were realized in a Precision XS Microplate Sample Processor (Biotek™). The isoniazid was dissolved in distilled water, as recommended by the manufacturer (Difco), and used as a standard drug. MTB $H_{37}$Rv ATCC 27294 was grown for 7–10 days in Middlebrook 7H9 broth supplemented with OADC, plus 0.05% Tween 80 to avoid clumps. Cultures were centrifuged for 15 min at 3,150 × *g*, washed twice, resuspended in phosphate-buffered saline, and aliquots were frozen at -80°C. After 2 days, an aliquot was thawed to determine the viability and the CFU after freezing. MTB $H_{37}$Rv ATCC 27294 was thawed and added to the test compounds, yielding a final testing volume of 200 µL with $2 \times 10^4$ CFU/mL. Microplates with serial dilutions of each compound were incubated for 7 days at 37°C, after resazurin was added to test viability. Wells that turned from blue to pink, with the development of fluorescence, indicated growth of bacterial cells, while maintenance of the blue color indicated bacterial inhibition[37,38]. The fluorescence was read (530-nm excitation filter and 590-nm emission filter) in a SPECTRAfluor Plus (Tecan®) microfluorimeter. The minimum inhibitory concentration (MIC) was defined as the lowest concentration resulting in 90% inhibition of growth of MTB[38]. As a standard test, the MIC of isoniazid was determined on each microplate. The acceptable range of isoniazid MIC is from 0.015 to 0.06 µg/mL[37,38]. Each test was set up in triplicate.

## RESULTS AND DISCUSSION

### Chemistry

The aza-stilbene derivatives **1**–**8** were synthesized by the classical method of imine formation involving condensation between 4-hydroxy-aniline with a variety of aromatic aldehydes in EtOH (Scheme 1). All compounds were characterized by $^{1}$H and $^{13}$C NMR, IR, and m.p. (Table 1), and were in accord with data in the literature[39,40,41,42,43].

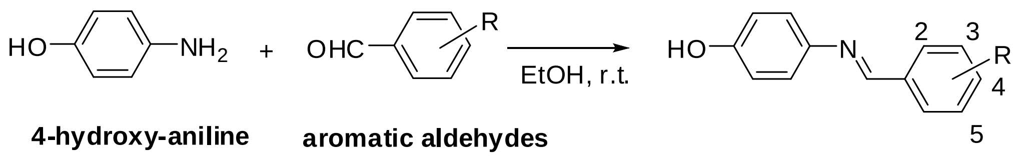

**1**: $R_2 = R_3 = R_4 = R_5 = H$
**2**: $R_4 = OH$
**3**: $R_2 = OH$
**4**: $R_4 = OH$, $R_5 = OMe$
**5**: $R_4 = OMe$
**6**: $R_3 = R_4 = R_5 = OMe$
**7**: $R_4 = NMe_2$
**8**: $R_4 = NO_2$

**SCHEME 1**. Synthesis of aza-stilbene derivatives **1**-**8**.

### Biology

- **Anti-MTB activity assay** — The *in vitro* anti-MTB activities of the aza-stilbene derivatives were tested against MTB $H_{37}Rv$ ATCC 27294 and the MICs are reported in Table 2. The MICs of compounds **1–5** and **7–8** were the same (15.6 µg/mL), and compound **6** was less active than the others compounds (31.25 µg/mL). This is the first report of activity against *in vitro* MTB infection in aza-stilbene derivatives and these results are comparable with pyrazinamide (MIC of 50–100 µg/mL), a “first”-line drug used in current therapy[44], and better than natural stilbene in assays against *Mycobacterium* species described in the literature.
- ***In vitro* structure activity relationship (SAR)** — As can be seen in Table 2, the results of the condensation between 4-hydroxy-aniline with a variety of aromatic aldehydes in EtOH showed similar MIC among the compounds **1–5**, **7,** and **8**. Then due to little difference between the results of the compound **6** with the others, indicate that the basic structure, the aza-stilbene skeleton, is fundamental to the good results presented by these molecules. It is interesting to note better MIC against MTB in aza-stilbene derivatives shown here than natural stilbene against *Mycobacterium* species[23,24,25]. It is noteworthy that the presence of three methoxy groups in compound **6** slightly decreased the activity. The only observation that can be made about this structure is the volume of the methoxy groups, since the presence of this group, compound **5**, does not affect the activity.

**TABLE 2**
***In vitro* Antiproliferative Activity of the Compounds 1–8 against MTB $H_{37}Rv$ ATCC 27294**

| Compound | MIC (µg/mL) |
|---|---|
| 1 | 15.6 |
| 2 | 15.6 |
| 3 | 15.6 |
| 4 | 15.6 |
| 5 | 15.6 |
| 6 | 31.25 |
| 7 | 15.6 |
| 8 | 15.6 |
| Pyrazinamide | 50–100 |

### CONCLUSION

This is the first report of activity against *in vitro* MTB infection in aza-stilbene derivatives. The results show that these compounds can be considered as promising anti-MTB agents, with anti-MTB activity comparable to pyrazinamide and better than natural stilbene tested with other *Mycobacterium* species. Further biological studies are required to show the efficacy and safety of these compounds *in vivo*, and to shed further light on the mechanism of the pharmacological action.

### ACKNOWLEDGMENTS

This study was supported by CNPq, FAPEMIG, Capes and Fundação de Amparo à Pesquisa do Estado de São Paulo (FAPESP), ref. Processes: 2008/10390-2 and 2009/06499-1.
